# The Impact of Chronic Suppurative Otitis Media with and without Cholesteatoma in Patients from Northeastern Romania

**DOI:** 10.3390/healthcare11010073

**Published:** 2022-12-27

**Authors:** Roxana Serban, Otilia Elena Frasinariu, Bianca Simionescu, Luminita Mihaela Radulescu, Bogdan Mihail Cobzeanu, Cristiana Filip, Ana Maria Laura Buga, Luiza Simona Pohaci Antonesei, Oana Raluca Temneanu

**Affiliations:** 1 Department of Morfo-Functional Sciences II, “Grigore T. Popa” University of Medicine and Pharmacy, 700115 Iasi, Romania; 2 Department of Mother and Child, “Grigore T. Popa” University of Medicine and Pharmacy, 700115 Iasi, Romania; 3 Department of Mother and Child, Pediatric Clinic nr 2, Crisan Street, Nr 3\5, University of Medicine and Pharmacy, “Iuliu Hatieganu”, 400012 Cluj-Napoca, Romania; 4 Department of Surgery II, “Grigore T. Popa” University of Medicine and Pharmacy, 700115 Iasi, Romania

**Keywords:** quality of life, chronic suppurative otitis media, cholesteatoma, COMQ-12

## Abstract

Quality of life is a widely used concept that tends to become an important part of clinical management. The present study performs an analysis of the impact of suppurative chronic otitis media with and without cholesteatoma on quality of life, using the COMQ-12 questionnaire. It was applied to a group of 40 healthy people and to 40 patients before surgery, and the answers to the questions were analyzed and correlated with socioeconomic factors. After the confirmation of the diagnosis based on clinical and imaging information, the patients completed the COMQ-12 questionnaire. It was observed that the chronic ear problems had negative impacts of varying degrees on daily and long-term activities. The evaluation and analysis of information can be used in setting therapeutic targets.

## 1. Introduction

The general concept of quality of life was developed for the first time after World War II [1]. In the field of health, quality of life was introduced as a feature in the 1960s [2], with health-related quality of life (HRQoL) being an indicator of an individual’s general state of health, including general information regarding the physical and the mental health of a person and the impact of health quality on their whole life. HRQoL is assessed by multiple indicators of the general state of health and physical and emotional health from an individual point of view [3,4].

The Chronic Otitis Media Questionnaire-12 (COMQ-12) was developed for the first time in 2014 by Phillips, who created an initial list of 33 questions, which was later narrowed down by removing questions that were similar [5].

Chronic suppurative otitis media (CSOM) can be classified into: CSOM without cholesteatoma and CSOM with cholesteatoma [6,7].

CSOM presents symptoms, such as otorrhea, local pain, hearing loss, that can lead to communication problems, impeding social interaction and resulting in a negative impact on the development of professional life [8].

The COMQ-12 questionnaire, as it stands today, was introduced 2014 and subsequently validated and translated into different languages, namely Dutch, Serbian, Russian, Portuguese, Turkish, Korean and Italian [5,9,10,11,12,13,14,15], allowing the evaluation of the impact of the disease on the patient from their own perspective. The Romanian version was developed in 2021 [16].

The aim of our study was to evaluate the quality of life in adult patients with CSOM using the COMQ-12 questionnaire.

## 2. Materials and Methods

The COMQ-12 questionnaire was applied to two groups.

The first group was the control, containing 40 volunteers. The inclusion criteria were as follows: age over 18 years, absence of a psychiatric pathology, native Romanian speakers and absence of a medical history of chronic otitis media.

The study group was formed of 40 patients diagnosed with CSOM with and without cholesteatoma, hospitalized in the Ear Neck and Throat Clinic of the Clinical Rehabilitation Hospital in Iasi, Romania. The questionnaire was completed before surgery. The inclusion criteria were as follows: patients native speakers of the Romanian language, without declared psychiatric disorders, age over 18 years, history of CSOM for at least 6 months, the existence of the clinical diagnosis of CSOM with or without cholesteatoma, the completion of the informed consent to participate in the study and the existence of the indication for surgical treatment.

The exclusion criteria from the study were as follows: incomplete responses of patients in the questionnaire, refusal to participate in the study and non-participation in the periodic evaluation.

The questionnaire contains 12 questions grouped into four categories, each with 6 answer options, on a scale from 0 to 5 points. The questions can be grouped as follows: Questions 1–7 refer to the severity of symptoms, Questions 8 and 9 refer to the impact of the disease on work and lifestyle, Questions 10 and 11 analyze the degree of accessibility to specialized medical services and Question 12 refers to the general impact of the condition on the patient.

The data obtained were centralized in an SPSS database, variant 18.0, and processed with suitable statistical functions at a significance threshold of 95%.

The ANOVA test was used to evaluate the descriptive statistical indicators: minimum, maximum, mean, median, standard deviation, standard error of the mean and variance. The F test (ANOVA) was used for the comparison in 3 or more groups of values with normal distributions

## 3. Results

The analysis of the study group, formed by patients with different forms of CSOM, from a descriptive point of view, highlights the preponderance of female cases (52.5%) compared to male cases. The gender ratio was 1.1/1.

With a variance of 35%, the age of the patients varied between 18–66 years, registering a mean value of 41.25 ± 14.25 years and a median of 39 years.

Analyzing the values obtained by gender, it should be noted that the mean age was higher in female patients than in male patients (45.52 vs. 36.53 years; *p* = 0.045) (Table 1); the minimum and maximum age were recorded in male patients (18 and 66 years, respectively).

By age group, in women, the largest age group (40–49 years) comprised 23.8% while in men it is the age group 30–39 years (42.1%), making the percentage differences statistically significant (*p* = 0.023), as can be seen in Table 2.

Regarding place of origin, the structure of the group by gender was homogeneous, with 57.9% of males and 47.6% of females from urban areas (*p* = 0.515).

According to studies, a high level of education was noted: 45% completed high school, 22.5% completed upper-secondary, 25% completed secondary and 7.5% completed primary school.

In patients with CSOM with cholesteatoma, the left ear was more frequently affected (60.7%), while CSOM without cholesteatoma was more common in the right ear (58.3%), but the percentage differences were not statistically significant (*p* = 0.267).

In the study group, the most used surgical technique was canal wall down (CWD) mastoidectomy (42.5%) in 17 patients, out of which 11 (64.7%) had CWD as initial technique and 6 (35.3%) required revision surgery after CWD. Canal wall up (CWU) with tympanoplasty was performed on 27.5% of patients.

When analyzing the answers to the first question, it can be seen that 45% of patients answered that otorrhea is a major inconvenience, which they can hardly cope with, but 17.5% of patients were not bothered at all by this symptom.

Regarding the second question, the case study showed that the most common symptom was an unpleasant smell in the ear, which 45.5% of patients could hardly cope with but 22.5% of patients were not bothered by at all.

In terms of the third question in the questionnaire, regarding the impact of hearing loss on the patient, 72.5% of respondents considered hearing problems to be a major inconvenience for the situation at home, 42.5% struggled to cope with the situation, and 10% of patients considered this symptom to be the worst thing affecting their lives.

In question 4, which analyzes the impact of hearing loss during conversation, 22.5% of patients had major difficulties participating in conversations and for 27.5% it was a major inconvenience, which they faced with difficulty, while 32.5% thought that it was also major but they felt able to cope with the situation.

Analyzing the answers to question 5, regarding local discomfort due to the ontological problem, 65% of patients considered the discomfort in/around the ear to be a major inconvenience, 25% found it difficult to cope with the situation, and 5% of patients considered this symptom to be the worst affecting their lives.

In Question 6, dizziness or loss of balance did not bother 47.5% of the patients surveyed, but 15% considered it a major risk that they do not face well and 7.5% considered it to have a major impact.

Out of the total number of patients surveyed, 52.5% were not bothered at all by noises in their ear, but for 22.5% this symptom represented a major inconvenience, althrough one which the patient was able to cope with.

For Items 1–7, a score was calculated, which varied from 0 (good state) to 35 (bad state). In the studied cases, the score for symptoms varied from 4 to 28, registering a mean level of 18.20 ± 6.68, which shows that the symptoms moderately affected well-being: 22.5% of patients reported a degree of mild impairment, 47.5% moderate and 30% severe.

In terms of comparison by gender (19.16 vs. 17.22; *p* = 0.395), age groups (18.10 vs. 18.32; *p* = 0.919) or place of origin (18.24 vs. 18.16; *p* = 0.970), the mean score for symptoms did not differ significantly from a statistical point of view (Table 3).

Severe impairment is more common in men (53.3%; *p* = 0.439) of ages under 40 (54.5%; *p* = 0.950) and in patients from urban areas (54.5%; *p* = 0.860).

Depending on the question, the average score for symptoms was significantly higher in patients with university and high school education, compared to those with primary or secondary education (21.90; 19.44 vs. 11.33; 16.67; *p* = 0,05).

Analyzing the answers to question 8, the ear impairment does not affect daily activities in 25% of patients; in 50% the daily performances was affected at least once a month.

Question 9 refers to the fear of developing local infections. Regarding this issue, 27.5% of patients failed to wash most days and 32.5% failed at least once a week, while only 2.5% failed to wash once in 6 months.

The answers to question 10, which refer to the degree of accessibility in medical services, show that the majority of patients went to the doctor for ear problems at least once a month (52.5%), but there were cases in which symptoms were addressed daily (10%) or weekly (15%).

Analyzing the answers to question 11, regarding the use of systemic or topical drugs for the treatment of the otological disease, the dominant answer was at least once a month (47.5%) but also every 3 months (30%).

For Items 8–11, a score was calculated which varied from 0 (good state) to 20 (bad state). In the cases studied, the score for symptoms ranged from 7 to 19, recording a mean level of 12.43 ± 2.90, which shows that the impact on lifestyle and medical services was moderately impaired: 57.5% of patients reported a moderate degree of impairment and 42.5% severe, as can be seen in Table 4.

Regarding comparison by gender (11.95 vs. 12.86; *p* = 0.328), age groups (12.42 vs. 12.43; *p* = 0.994) or place of origin (11.86 vs. 13.05; *p* = 0.197), the mean score for lifestyle impact did not demonstrate statistically significant differences.

Severe impairment is more common in women (64.2%; *p* = 0.182) at ages under 40 (58.8%; *p* = 0.490) and in patients from rural areas (52.9%; *p* = 0.553).

Depending on the question, the average score for impact did not register significant differences in terms of educational level (*p* = 0.175).

Regarding the type of disease, the impact score was moderate in 64.3% of patients with CSOM with cholesteatoma, while the impact score was severe in 58.3% of patients with CSOM without cholesteatoma (*p* = 0.186).

For the last question of the questionnaire, which assesses the general impact on the patient, the case study found that most frequently, otological problems are a moderate inconvenience for 32.5% of patients and a major inconvenience for 60%, and around 30% find it hard to deal with.

## 4. Discussion

Quality of life in health is described as the perception of an individual related of the impact of a disease and its treatment from a physical, mental and social point of view. Quality of life assessments include subjective and objective aspects [17].

Knowing the factors that influence the quality of life is useful in the analysis of data related to CSOM. the degree of hearing loss and persistent local symptoms are associated with a low level of quality of life [5]. Prabhu et al. showed that otic disease has a negative impact on the patients’ quality of life [17].

The first question in the questionnaire assessed the impact of a primary local symptom, namely otorrhea, which was reported a major inconvenience for almost half of the patients. There were also some patients who were not bothered at all by this symptom. We can thus say that one of the dimensions of HRQoL is affected, namely physical health [17]. There are institutions that routinely use quality of life assessment tools to assess the patient’s conditions before, during, and after treatment [18].

Regarding the second question, which refers to an unpleasant smell in the ear, it was found that this aspect was a major inconvenience, which patients could hardly cope with; a result that is supported by almost half of the participants in the study. In equal percentages, patients perceived this factor as a major inconvenience, but one which they felt able to cope with, or a moderate or small inconvenience. Some patients reported that they were not bothered at all by this local symptom. Usually, the smell is determined by a bacterial infection, with the bacteria being staphylococci (most common in general infections also) or necrotic tissue. The large number of affected patients shows the major impact on physical and mental well-being. Interaction and interpersonal relationships can also be influenced [19,20]. In his study, Bakir identified an increase in anxiety, depression and interpersonal sensitivity in patients with CSOM, in addition to a decrease in their level of general health perceptions and their degree of social functionality [21].

The next question in the questionnaire, the one regarding the impact of hearing impairment on the patient, demonstrated that many interviewees considered hearing problems at home to be a major inconvenience. There were also cases where hearing loss was only a small or moderate inconvenience. The impact on quality of life is observed in all categories of patients, regardless of gender, age and environment, varying only in intensity.

The fourth question analyzes the impact of hearing loss in a conversation. Only a small percentage of those interviewed were not affected at all by this aspect. The difficulty of coping in a conversation has an impact on mental and social health, influencing communication, the ability to integrate socially and interrelationships but also cognitive ability and concentration.

The fifth question of the questionnaire in the Romanian COMQ-12 refers to local discomfort due to the otological problem, with more than a half of the patients considering the discomfort in/around the ear a major inconvenience which affects their daily activities, with 25% struggling to cope with the situation and just a small number of the patients considering this symptom the worst thing affecting their life. However, there were also cases in which the local problem was considered a moderate to minor inconvenience. We can thus consider that the otological problem is an important disturbing factor which cannot be ignored or neglected.

Question 6 refers to the impact of balance disorders on the patient. This aspect did not affect most of the patients surveyed. Only a few considered it a major risk that they could not face with or considered it to have a major impact on the activities carried out during a day. In the study conducted by Fonseca et al., balance has the greatest impact on the study group [12].

Question 7 of the questionnaire assesses tinnitus. About half of the patients interviewed were not bothered by noise in their ear. Some patients reported tinnitus as a major inconvenience but were not significantly affected it. A small number of patients had real difficulties in managing the problems caused by tinnitus. Fonseca noted that tinnitus is one of the most significant local problems caused by CSOM [12].

For some people with tinnitus, the perceived effects of this symptom have a negative impact on the various dimensions of quality of life. For many people who complain of tinnitus, there are medical, audiological or psychological causes that determine the symptoms. The severity of tinnitus is related to the perceived attitude of the individual in relation to other conditions. The prevalence of this symptom is much higher than its actual declaration. This shows that, for most people, it goes away or becomes unnoticeable background noise. When other psychosomatic stressors are present, tinnitus becomes annoying. The most common psychological changes that can be caused by tinnitus are difficulty concentrating, irritability and sleep disturbances. These may be related to mood disorders [22,23].

Half of those surveyed had a moderate quality of life and just one third had severe impairment. Analyzing the onset of local symptoms by gender, age group and place of origin, the mean score value for symptoms did not differ statistically significantly, suggesting that local symptoms were perceived similarly by patients. This study thus addresses one of the four dimensions of quality of life related to health, namely physical health involving somatic perception and the impact of disease symptoms.

The impact on daily activities was high, with half of those who completed the questionnaire declaring that their daily business was disrupted at least once a month. A small percentage of the studied group reported an almost daily impairment of daily activities. Thus, the otological condition negatively influences the quality of life of each person. Obstructing daily routined leads to a decrease in self-care capacity.

Daily hygiene, one of the most basic needs of the patient, as defined by Henderson, is affected by otological pathology: a third of patients failed to wash most days, and a small number at least once a week. A small number of the patient failed to wash less than once in 6 months.

Personal hygiene is a key to primary health care. Inadequate hygiene causes the spread of disease, decreased work capacity and poor quality of life [24]. Thus, the inability to perform personal grooming due to otological disease leads to a decrease in self-confidence and an impairment of daily activities.

Question 10 of the Romanian version of the COMQ-12 questionnaire refers to the degree of access of medical services. Almost half of the patients went to the doctor for otological problems at least once a month. A small percentage of only 2.5% went to the doctor less than once every 6 months. This was directly proportional to the access to medical services. The large number of cases that accessed medical services in order to treat/cure the otological condition indicated increased medical costs.

For question 11, “How often should you take medication (including local drops) for your ear problem?”, half of the patients stated that they used medication at least once a month, a third at least once every 3 months and a very small number took medication at least once every 6 months. Thus, local treatments that must be administered constantly decrease an individual’s quality of life.

For questions 8 to 11, a score was calculated, indicating that the impact on lifestyle and medical services was moderate. Half of the patients reported a moderate degree of impairment or a severe degree of impairment. When analyzing the answers obtained according to gender, age groups or place of origin, it can be seen that these factors do not show statistically significant differences. In the present study, the most severe impact was more frequent in women under the age of 40 years and in patients from rural areas. Depending on the level of studies, the average impact score did not show significant differences.

The last question of the questionnaire, which assessed the general impact on the patient, found that the most common otological problems are a major inconvenience for more than a half of those interviewed. All patients were affected by the otological problem. Those with a high score on this question had the highest risk scores for symptoms or lifestyle impact. Thus, the general impact created by the otological disease on the patient tends to be a major inconvenience, affecting all dimensions of quality of life.

Centralizing the data obtained by applying this questionnaire, we can say that demographic aspects do not significantly affect the deterioration of well-being caused by otological problems. The source environment is involved in the determinism of demoralization caused by 60% of otological problems. This is associated with economic resources, education and access to health services. There are studies that show the association between socioeconomic status and physical and mental health. Risk scores influenced by certain symptoms or lifestyle choices, alongside the deterioration of an individual’s mental state [25].

## 5. Conclusions

This questionnaire is designed to provide as much information as possible concerning the symptoms of patients and how they are affected. This study will help clinicians to choose an appropriate management strategy that is consistent with the patient’s expectations and needs.

Analyzing the values obtained after questionnaire completion, we were able to observe the negative impact of the otological disease, regardless of its type, on various aspects of patients’ quality of life.

## Figures and Tables

**Table 1:** Descriptive statistical indicators of age (years).

Gender	N	Mean	SD	Std. Error	CI		Min	Max	pANOVA Test
					−95% CI	+95% CI			
**Male**	19	36.53	14.07	3.23	29.74	43.31	18	66	0.045
**Female**	21	45.52	13.32	2.91	39.46	51.59	22	63	
**Total**	40	41.25	14.25	2.25	36.69	45.81	18	66	

**Table 2:** Structure group by age group and gender.

Age Group	Male		Female		Total	
	*n*	%	*n*	%	*n*	%
<20 years	2	10.5%	0	0.0%	2	5.0%
20–29 years	4	21.1%	3	14.3%	7	17.5%
30–39 years	8	42.1%	4	19.0%	12	30.0%
40–49 years	1	5.3%	5	23.8%	6	15.0%
50–59 years	3	15.8%	5	23.8%	8	20.0%
60–69 years	1	5.3%	4	19.0%	5	12.5%

**Table 3:** Descriptive statistical indicators of the risk scores for symptoms.

Characteristics	N	Mean	SD	Std. Error	CI		Min	Max	pANOVA Test
					−95% CI	+95% CI			
Total	40	18.20	6.68	1.06	16.06	20.34	4	28	
**Gender**									
Male	19	19.16	7.33	1.68	15.63	22.69	4	28	**0.395**
Female	21	17.33	6.09	1.33	14.56	20.10	7	26	
**Age group**									
<40 years	21	18.10	7.17	1.56	14.83	21.36	4	28	**0.919**
≥40 years	19	18.32	6.29	1.44	15.28	21.35	7	27	
**Place of origin**									
Urban	21	18.24	6.62	1.45	15.22	21.25	7	28	**0.970**
Rural	19	18.16	6.92	1.59	14.82	21.49	4	28	
**Studies**									
Primary school	3	11.33	3.21	1.86	3.35	19.32	9	15	**0.050**
Gymnasium	10	16.67	5.78	1.83	17.76	26.04	11	28	
High school	18	19.44	6.06	1.43	13.65	19.68	7	27	
University education	9	21.90	7.58	2.53	13.61	25.27	4	28	

**Table 4:** Descriptive statistical indicators of the impact score.

Characteristics	N	Mean	SD	Std. Error	CI		Min	Max	pANOVA Test
					−95% CI	+95% CI			
Total	40	12.43	2.90	0.46	11.50	13.35	7	19	
**Gender**									
Male	19	11.95	3.27	0.75	10.37	13.53	7	19	**0.328**
Female	21	12.86	2.52	0.55	11.71	14.00	8	17	
**Age group**									
<40 years	19	12.42	2.80	0.64	11.07	13.77	8	17	**0.994**
≥40 years	21	12.43	3.06	0.67	11.04	13.82	7	19	
**Place of origin**									
Urban	21	11.86	2.99	0.65	10.50	13.22	7	17	**0.197**
Rural	19	13.05	2.74	0.63	11.73	14.37	8	19	
**Studies**									
Primary school	3	13.00	1.00	0.58	10.52	15.48	12	14	**0.175**
Gymnasium	10	13.70	3.16	1.00	11.44	15.96	8	19	
High school	18	11.33	2.79	0.66	9.95	12.72	8	17	
University education	9	13.00	2.78	0.93	10.86	15.14	7	16	

## Data Availability

The data presented in this study are available upon request from the corresponding author. The data are not publicly available due to respondent privacy.
